# Enteral Nutrition Support and Short-Term Weight Outcomes in Pediatric Avoidant/Restrictive Food Intake Disorder and Anorexia Nervosa During Inpatient Medical Stabilization: A Retrospective Cohort Study

**DOI:** 10.3390/nu18162709

**Published:** 2026-08-19

**Authors:** Elizabeth M. Noonan, Jason M. Doherty, Amanda Ferrito, Darshwana Arunprasath, Alexandra Bush-Kaufman, Grace E. Monterubio

**Affiliations:** 1 School of Medicine, Saint Louis University, Saint Louis, MO 63104, USA; jason.doherty@health.slu.edu (J.M.D.);; 2 Department of Pediatrics, SSM Health Cardinal Glennon Children’s Hospital, 1465 S. Grand Blvd, Saint Louis, MO 63104, USA; 3 Department of Pediatrics, Medical College of Wisconsin, Milwaukee, WI 53226, USA

**Keywords:** malnutrition, eating disorder, nasogastric tube

## Abstract

**Background/Objective:** Avoidant/Restrictive Food Intake Disorder (ARFID) is increasingly recognized among hospitalized pediatric patients with malnutrition; however, optimal nutritional management strategies remain poorly defined. Many programs utilize protocols developed for anorexia nervosa (AN). This study examined patterns of nasogastric tube (NGT) use and weight trajectories in pediatric patients with ARFID and AN/atypical AN (AAN) treated under the same inpatient refeeding protocol. **Methods:** We conducted a retrospective study of pediatric patients admitted for medical stabilization of malnutrition at a tertiary care children’s hospital (*n* = 160; ARFID subgroup *n* = 34). Data included NGT utilization and weight change during hospitalization. Longitudinal change in BMI z-scores during hospitalization was modeled using multivariable analysis. **Results:** Patients with ARFID were more likely to require NGT than those with AN/AAN (*p* < 0.001), despite similar BMI z-scores at admission and discharge. Patients with ARFID requiring NGT were younger and had lower BMI z-scores at admission than ARFID patients without NGT (*p* < 0.05). **Conclusions:** In this study, pediatric patients with ARFID undergoing inpatient nutrition rehabilitation achieved comparable weight gain during hospitalization to patients with AN/AAN but required higher enteral nutrition support to do so. Among patients with ARFID who required NGT support, anthropometric measures suggested higher baseline nutritional severity and likely confounding by indication. Because NGT use was determined clinically rather than randomly assigned, the observed association between NGT use and higher weight gain should not be interpreted as evidence of a causal treatment effect. Interpretation is also limited by variability in clinical practice and the relatively small ARFID subgroup. This study supports diagnosis-specific considerations during medical stabilization and underscores the need for further ARFID-specific protocols and research.

## 1. Introduction

Avoidant/restrictive food intake disorder (ARFID) is a heterogeneous condition introduced in the Diagnostic and Statistical Manual-5 (DSM-5) in 2013 and revised in 2022. Diagnosis of ARFID is typically made when patients present with growth failure (low appetite/lack of interest subtype), sensory sensitivity-related food restriction leading to nutrient deficiencies or psychosocial distress (sensory sensitivity subtype), or acute weight loss following an adverse event like illness, choking, or traumatic events (fear of aversive consequences subtype) [1,2]. In contrast to anorexia nervosa (AN) and atypical anorexia nervosa (AAN), these patients do not have a drive for thinness or body image distortion [1,3,4]. Many patients with ARFID exhibit multiple contributing factors underlying restrictive intake, with gastrointestinal symptomatology and anxiety being the most common comorbidities [5,6,7,8,9,10,11,12]. Early diagnosis and treatment are likely to improve patient outcomes and prevent consequences of malnutrition, but the heterogeneity of this disorder complicates the research and standardized treatment of ARFID [3,6,13,14,15,16].

ARFID is increasingly recognized as a significant cause of pediatric malnutrition, growth impairment, and medical instability requiring hospitalization. Despite growing recognition of ARFID, much of the available treatment literature consists of small observational studies, case series, and emerging behavioral interventions, leaving substantial uncertainty regarding optimal inpatient nutritional management practices [17,18]. Recent literature has demonstrated that children and adolescents with ARFID frequently exhibit significant macro- and micronutrient inadequacies, including deficiencies in protein, vitamins, and minerals [19,20]. In medically compromised patients, nutritional rehabilitation is essential to restore growth, correct nutrient deficiencies, and reverse the complications of malnutrition [11,21,22,23,24]. While inpatient refeeding protocols developed for anorexia nervosa are commonly adapted, it remains unclear whether these approaches adequately address the unique feeding challenges associated with ARFID [11,14,21,22,23]. Enteral nutrition delivered through a nasogastric tube (NGT) is used when oral intake is insufficient to meet nutritional goals; however, evidence regarding the role of NGT supplementation during inpatient nutritional rehabilitation of youth with ARFID remains limited [25,26,27,28]. This study aimed to compare the clinical characteristics, enteral nutrition support requirements, length of stay, and weight outcomes among patients with ARFID and AN/AAN treated using an inpatient eating-disorder refeeding protocol at a pediatric tertiary care center.

## 2. Methods

### 2.1. Ethics Statement

This work was granted approval, and participant consent was waived/exempt by the Saint Louis University Institutional Review Board (Protocol #33633; approved 21 December 2023). The requirement for informed consent was waived because the study involved a retrospective review of existing records that were deidentified prior to analysis and posed minimal risk to participants.

### 2.2. Participants

Patients diagnosed with AN, AAN and ARFID, 9 to 20 years of age, admitted for medical stabilization and treated using the eating disorder refeeding protocol from January 2017 to June 2023 were included (*n* = 160; AN/AAN = 126, ARFID = 34). Patients were excluded if they did not complete the protocol, if they needed partial or total parenteral nutrition or nasoduodenal tube use (modification to the standard protocol; *n* = 9). Only the initial hospitalization for medical stabilization was analyzed.

### 2.3. The Refeeding Protocol

At the described medical center, patients diagnosed with ARFID are treated using an inpatient refeeding protocol initially developed for patients with AN. The goals of treatment are to achieve an initial weight gain and the normalization of biochemical markers [29]. Admission eligibility is based on the Society of Adolescent Medicine indications for inpatient admission in adolescents with malnutrition from restrictive eating disorders [30].

The multidisciplinary team consists of a pediatric hospitalist physician, adolescent medicine physician, nursing staff, registered dietitian, pediatric psychologist, child life specialist, music therapist, physical therapist, inpatient schoolteacher, social worker, and child psychiatrist as needed. The protocol includes safety measures (activity limitations, laboratory and vital sign monitoring, bedrest orders, bathroom supervision), psychosocial protective measures (phone and internet/media limitations, school requirements, visitor guidelines), and nourishment guidelines (a stepped meal plan created by the registered dietitian with input from the patient and caregivers).

For ARFID, some exceptions were made to psychosocial protective measures (most commonly, allowing use of personal electronic devices while in their room). Safety measures were consistently upheld, regardless of the cause of malnutrition, due to the risk of secondary effects (hypotension, falls, heart arrhythmias, refeeding syndrome, etc.) [28,30]. Patients underwent daily laboratory monitoring during caloric advancement. Oral electrolyte supplementation was provided as needed for minor abnormalities identified on refeeding laboratory monitoring. No significant safety concerns related to refeeding were identified, and no patients required escalation of care. Weight was obtained daily using standardized protocol procedures with minimal clothing or in hospital gowns and after first morning void when feasible. During nutrition consultation, patients with AN are instructed to include at least half of the food preference sheet, while patients with ARFID are given more liberty with food preferences as long as all food groups are met [19]. Patients attended supervised group meals with standardized time limits followed by “sit time.” The pediatric psychologist and inpatient nurses trained in family-based treatment (FBT) and cognitive behavioral therapy (CBT)-based behavioral encouragement approaches are present to support patients and try to reduce the need for supplementation [14,31,32]. Nutrition therapy started at 1200–1400 kcal for the majority of patients during the study time frame. Caloric advancement followed a standardized protocol regardless of NGT use and typically increased by approximately 200 kcal/day until goal nutrition was achieved (with modification since the end of the study timeframe to start at higher calorie levels) [33].

A meal replacement shake is provided for any food not completed in the time allotted (30 min) at a 1:1 calorie ratio. Patients are given 15 min to drink the meal replacement shake. An NGT is used to deliver the meal replacement if the patient cannot finish the plated food or meal replacement by mouth within the allotted protocol timeframe (30 min for plated meal, followed by 15 min for meal replacement) [14,26]. The decision to leave the NGT in place versus removing after supplementation was determined by the multidisciplinary team based on the dependence on enteral supplementation, tolerance of the NGT placement, and the eating disorder diagnosis. For example, if anxiolytic medications were required to place the NGT or the patient required use of NGT with consecutive meals, then it was left in place until no longer needed for at least 24 h [22]. In August 2021, the team agreed upon a default of leaving the NGT in place for all patients with ARFID once it was required. Once a NGT was needed, if the daily caloric escalation or bolus infusions of the meal replacement were not tolerated (vomiting or refusal), overnight feed infusion was considered. The daily caloric escalation remained consistent regardless of NGT exposure.

This protocol is similar to previously published inpatient refeeding protocols but differs in these aspects: a child life specialist is engaged for support, therapy is provided weekly, and caregivers are not engaged with meals until discharge teaching is completed. Patients are discharged once they demonstrate tolerance by mouth and weight gain on the goal meal plan, vital signs and laboratory tests are stable, caregivers have received discharge teaching, and an outpatient treatment plan is created. Discharge teaching includes psychoeducation about FBT (for ARFID or AN) and the meal plan, followed by therapeutic family meals led by the psychologist.

### 2.4. Data Sources

Patient demographics, length of hospital stay, weights, BMI, tube-related data, and encounter diagnoses were extracted from the Epic Clarity database hosted on Microsoft SQL Server Management Studio (Epic Systems Corportation, Verona, WI, USA). Non-discrete data (eating disorder diagnosis, comorbid conditions, degree of malnutrition on admission, NGT use, discharge medications, and discharge destination) were extracted via retrospective chart review.

Patient charts were reviewed by medical student abstractors (AF, DA). Charts that were unclear for any variables were flagged for review by the adolescent medicine physician (EN). An overlap of 31 patients between the two raters was used to estimate the interrater reliability: before the exclusion criteria were applied, the raw agreement was 84% (Cohen’s κ = 0.773, *p* < 0.001); after the exclusion criteria were applied (blind to interrater reliability), the reliability was measured at 100% (κ = 1.00, *p* < 0.001).

Eating disorder diagnoses were based on psychology, adolescent medicine, and psychiatry assessments. The degree of malnutrition was identified using the BMI z-score. Charts not meeting the z-score criteria were reviewed by a registered dietitian (AB) and assigned a degree of malnutrition using additional anthropometric and intake data [34]. Nasogastric feeding was documented as a categorical variable with the options of no use, bolus infusion, overnight infusion, or both, and NGT removed or left in place.

### 2.5. Statistical Analysis

Analyses were conducted in R version 4.4.3 [35,36,37]. This retrospective observational study was reported in accordance with the Strengthening the Reporting of Observational Studies in Epidemiology (STROBE) guidelines. Sample characteristics were analyzed via t-test for continuous variables and chi-squared test for categorical variables. BMI values were transformed to age-adjusted z-scores using the Extended CDC BMI-for-age Growth Charts for Children and Adolescents [38,39]. The change in BMI z-scores was modeled via Generalized Linear Mixed Effects Modeling (GLMM), with a random intercept for patient. For patients placed on NGT, the day patients were first placed on NGT was used as t = 0 to more accurately estimate the effect of NGT by only including the weight change after the decision for NGT intervention. The day of admission was used as t = 0 for all other patients. Due to small daily changes in BMI, the time was expressed in weeks to aid interpretability (e.g., 4 days ≈ 0.57 weeks). Age at admission, sex, patient group (AN/AAN vs. ARFID), depression diagnoses, anxiety diagnoses, obsessive compulsive disorder diagnosis, and any NGT use were included alongside time (weeks) from admission to model BMI over the course of the inpatient encounters. The BMI z-scores at admission were also included to account for baseline weight differences. Three-way interaction terms between time, group and NGT plus lower-level interactions, were included to assess the differential longitudinal effects on patient groups. The conditional R^2^ (R^2^c) was 0.92, indicating a good model fit. The model diagnostics did not reveal any critical issues with the model fit (Variance inflation factor, VIF, <4; mild deviation from normal for residuals and mild heteroscedasticity). The inclusion of three-way interactions was supported by both Akaike and Bayesian information criteria when compared with the less complex model.

## 3. Results

Table 1 shows no difference in age, sex or race between the ARFID and AN/AAN groups. The BMI at admission and discharge were similar between groups, but patients with ARFID had a lower weight at admission and discharge (SMD = 0.42, *p* < 0.05). Patients with ARFID were more likely to require NGT (SMD = 0.78, *p* < 0.001), had lower rates of depression (SMD = 1.08, *p* < 0.001), and higher rates of phobia relating to vomiting or choking (SMD = 0.97, *p* < 0.001). There were no other differences between comorbid conditions, including anxiety (Table 2). Antihistamines (*p* = 0.051) and acid reducers (*p* = 0.052) had marginally higher prescription rates for ARFID at discharge, while selective serotonin reuptake inhibitors (SSRIs) and antipsychotic prescribing did not differ between the patient groups. No clinically significant safety concerns related to nutritional rehabilitation were identified.

**Figure 1:**
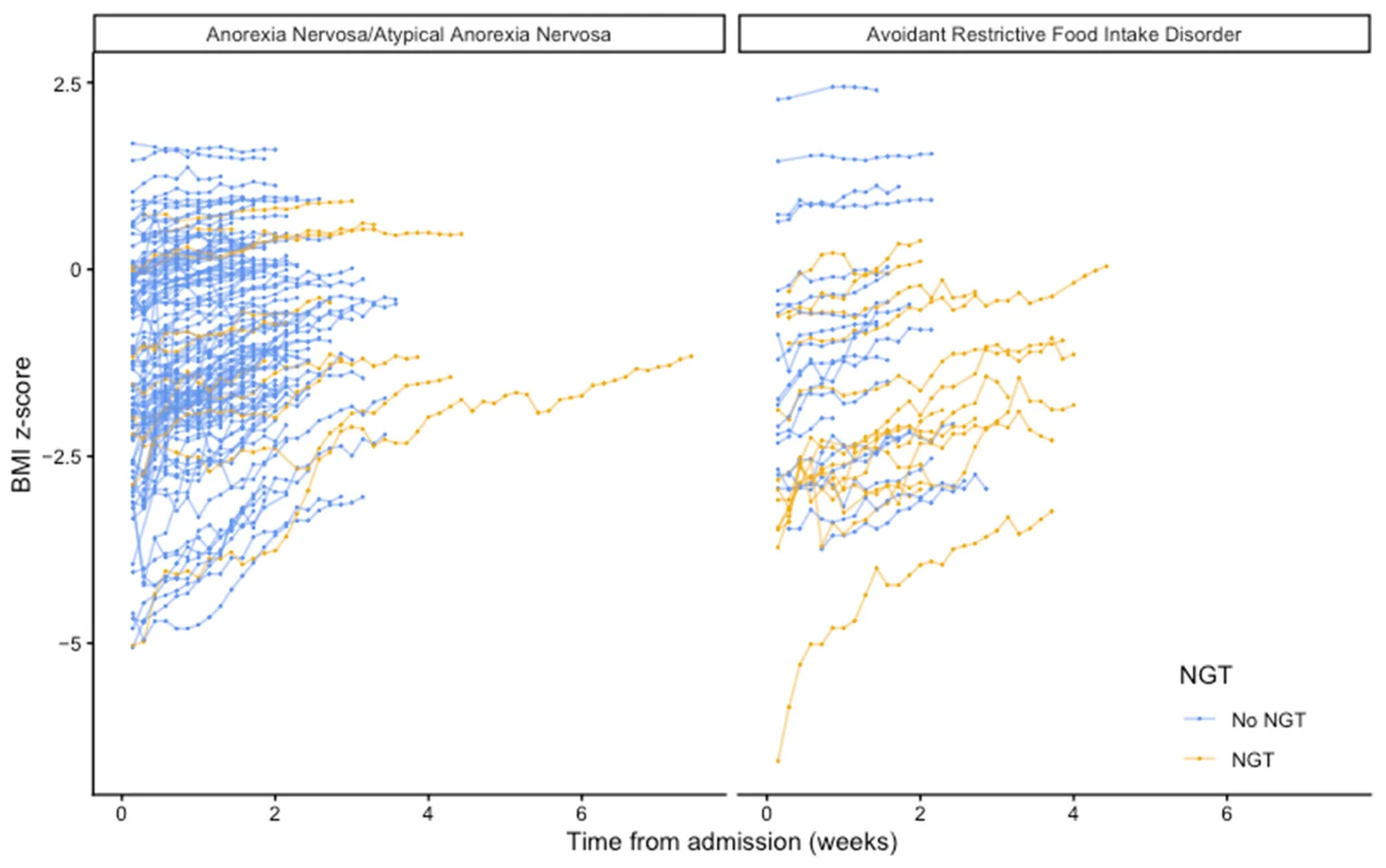
BMI z-score change over time for patients with AN/AAN and ARFID by NGT use. Sample sizes: AN/AAN without NGT (*n* = 114), AN/AAN with NGT (*n* = 12), ARFID without NGT (*n* = 20), ARFID with NGT (*n* = 14).

**Figure 2:**
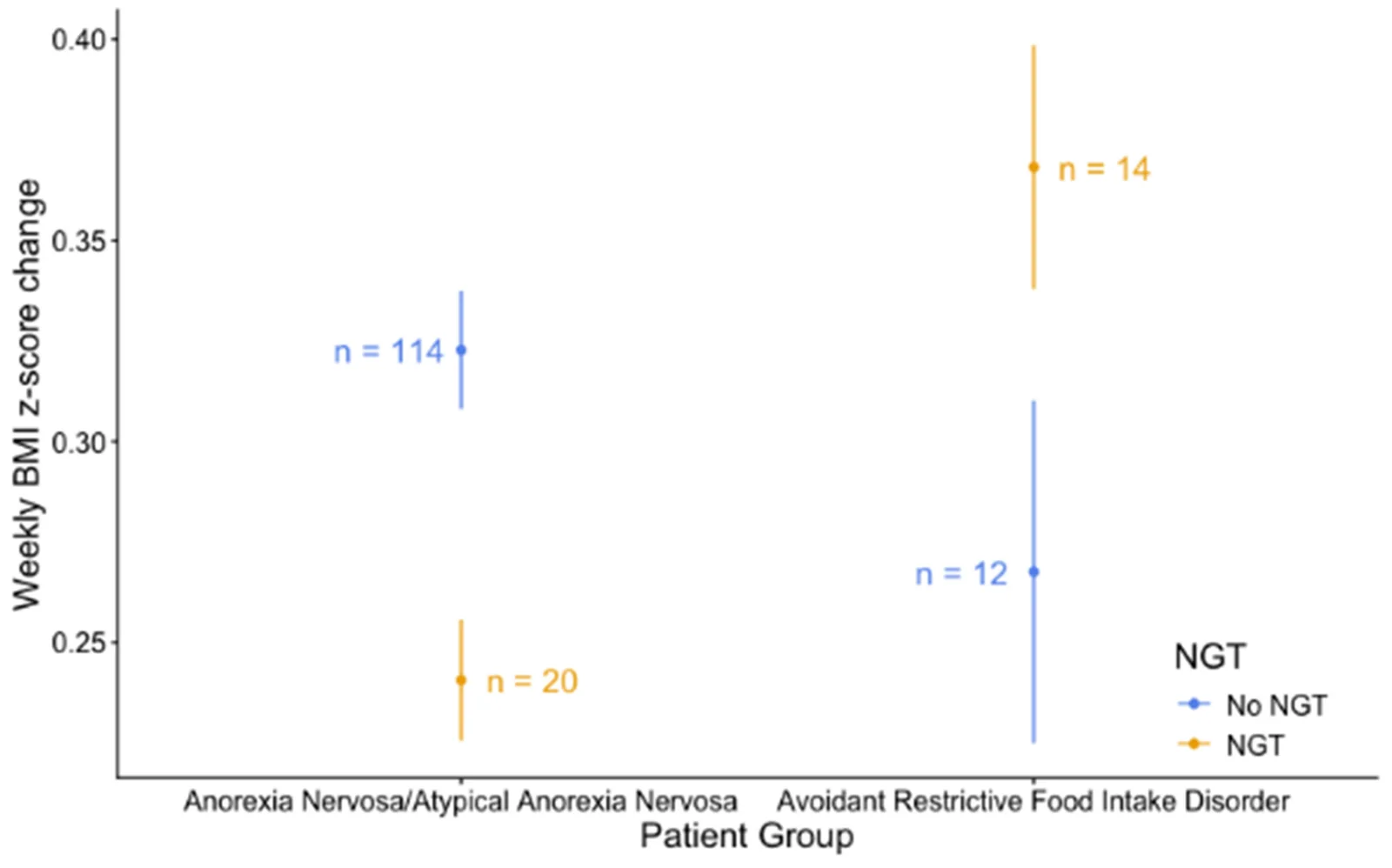
Estimated marginal effects of weekly rate of change in BMI z-score for patients with AN/AAN and ARFID stratified by NGT use. Error bars indicate 95% confidence intervals.

Figure 1 shows the BMI z-score change following admission, split by patient group and colored based on whether a patient was placed on NGT during their admission. The BMI analysis aimed to determine whether NGT use was associated with differential weight gain among patients with ARFID while controlling for covariates and whether this association differed from that observed in the AN/AAN group. This analysis identified an interaction between time, group and NGT (*p* < 0.001). Figure 2 shows marginal slope estimates of the weekly changes in BMI z-scores across different combinations of patient group and NGT status: ARFID patients who required NGT support demonstrated higher rates of weight gain than ARFID patients who did not require NGT support, whereas the opposite pattern was observed in the AN/AAN group. The model also included a significant effect of the BMI z-score at admission (*p* < 0.05) and a significant interaction between the baseline BMI and time (*p* < 0.001), indicating that patients admitted with higher BMI z-scores at baseline gained weight at a slower pace. Calculated using the model summarized in Table 3, the average marginal effect of NGT for ARFID patients was estimated at a 0.37 unit increase in z-score per week (95% CI 0.34, 0.40) compared to 0.27 (95% CI 0.23, 0.31) without. Conversely, for AN/AAN patients, the weight gain was higher for patients without NGT (0.32 increase in z-score, 95% CI 0.31, 0.34) than for patients with NGT (0.24, 95% CI 0.23, 0.26).

**Figure 3:**
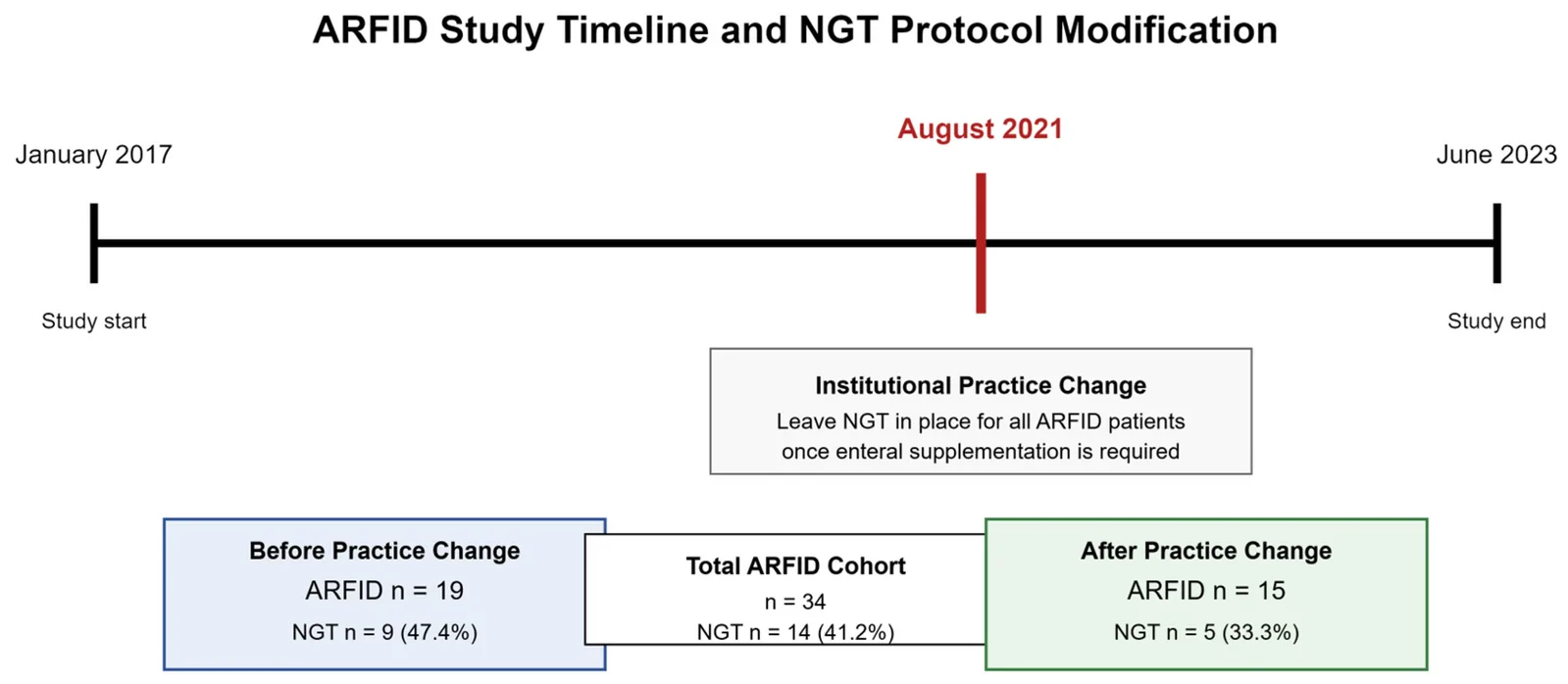
Timeline of ARFID patient inclusion, NGT utilization, and implementation of the August 2021 nasogastric tube (NGT) protocol modification. Nineteen patients with ARFID were hospitalized before the August 2021 practice change (9 requiring NGT support), and 15 were hospitalized afterward (5 requiring NGT support).

Table 4 shows an aggregate data comparison of the ARFID groups (with and without NGT use): the ARFID group with NGT use was younger (*p* < 0.05), had a lower BMI z-score at admission (*p* < 0.05), a longer length of stay (*p* < 0.001), and larger BMI z-score change from admission to discharge (*p* < 0.05). Of the 34 patients with ARFID included in the study, 19 (NGT = 9) were admitted prior to the August 2021 practice change and 15 after (NGT = 5) (Figure 3).

## 4. Discussion

In this cohort of youth with restrictive eating disorders hospitalized for medical stabilization, patients with ARFID achieved a weight gain comparable to patients with AN/AAN but required substantially higher use of NGT supplementation. NGT use was associated with higher weight gain among patients with ARFID; however, those requiring NGT support also demonstrated higher nutritional impairment at admission. These findings are consistent with the prior literature, suggesting that inpatient management of ARFID frequently requires more reliance on enteral nutrition, and highlight important differences in nutritional rehabilitation needs between ARFID and AN/AAN [11,20,27,28].

The ARFID and AN/AAN groups were similar with respect to age, sex, race, and admission BMI. Patients with ARFID can present across a broad range of BMI values, which supported the inclusion of both AN and AAN in the comparison group [2,11,15,27,28,40]. In addition, we expect BMI diversity within ARFID to continue to increase following the 2022 DSM-5 Text Revision, which permits diagnosis in the presence of psychosocial dysfunction impairment even without growth failure. Consistent with previous reports, patients with ARFID demonstrated a distinct comorbidity profile characterized by lower rates of depression and higher rates of phobia-related symptoms. Phobia-related presentations may reflect fear-of-aversive consequences in ARFID patients, which is commonly represented among hospitalized cohorts [3,8,22,27,41]. Contrary to several prior studies, we did not observe increased rates of gastrointestinal symptoms, anxiety, or autism spectrum disorder, potentially reflecting the limited statistical power or increasing heterogeneity among patients receiving an ARFID diagnosis [1,3,4,8,11,42,43].

ARFID patients did not differ from AN/AAN patients in terms of demographics, malnutrition, or BMI at admission, but they were more likely to require enteral nutrition support to achieve comparable weight gain. These findings support diagnosis-specific differences in nutritional rehabilitation requirements between patients with AN/AAN and ARFID. The higher need for enteral nutrition support likely reflects the core clinical features of ARFID, including limited dietary volume, restricted food variety, sensory sensitivities, and fear-based avoidance. These factors may impede progression through standardized meal-plan advancement despite the similar medical need for nutritional rehabilitation. Enteral nutrition may facilitate medical stabilization when oral intake is inadequate; however, it does not address the underlying drivers of ARFID. Prolonged dependence on enteral feeding may reduce opportunities for oral food exposure and theoretically reinforce restrictive eating behaviors [9,10,44,45]. Because many patients with ARFID experience sensory sensitivities and aversive eating-related fears, clinicians must balance the benefits of improved caloric delivery against the potential for increased distress and treatment dependence [1,10,22].

Patients with ARFID and NGT experienced a larger change in BMI z-score compared to patients with ARFID without NGT use; however, the discharge BMI z-scores were similar regardless of NGT exposure. These findings suggest that NGT use may be associated with achievement of nutritional goals among patients with ARFID experiencing difficulty meeting caloric needs orally. The observed relationship between NGT use and higher weight gain likely reflects confounding by indication, as patients with ARFID requiring NGT support were younger (consistent with other studies [25,26]) and presented with higher nutritional impairment. Other studies have shown slower weight gain in patients with a history of NGT use, which was observed in the AN plus NGT group [43]. Because the discharge criteria included full oral intake, patients with ARFID requiring NGT had longer lengths of stay. Nevertheless, interaction analyses suggest that the differences in weight trajectory were not solely attributable to hospitalization duration.

Several important limitations should be considered. This was a single-center retrospective study susceptible to residual confounding and limited generalizability. The findings of this study should also be interpreted within the context of its relatively small ARFID sample size (*n* = 34). NGT exposure was determined clinically rather than randomly assigned, and a practice change introduced in August 2021 altered NGT management among patients with ARFID. While the caloric advancement remained consistent regardless of NGT exposure, the sample size was insufficient to evaluate outcomes before and after implementation of this practice change. The small sample size also limited our ability to model the ARFID group in isolation for subgroup/sensitivity analyses. Together, these factors limit the causal interpretation of associations between NGT use and weight gain. In addition, detailed data regarding NGT duration, timing, and proportion of nutrition delivered enterally were not available to permit a time-dependent analysis, and the available sample is likely too small and the time period too short to have sufficient statistical power to model NGT use as a time-varying component [46]. We have restricted the analysis of weight gain on NGT to only the period after the first placement, to more accurately estimate the effect of the intervention. Future studies should examine NGT exposure longitudinally to better characterize its relationship with weight restoration.

During the study timeframe, the meal plans were routinely started at low calorie levels, which lengthened the hospitalization for all groups. Because the discharge criteria required achievement of adequate oral intake and medical stability, the length of stay and weight gain were not entirely independent outcomes. Readers should interpret the findings with awareness of these potential sources of bias. Long-term outcomes after discharge were not available; therefore, the impact of inpatient weight gain, NGT use, and hospitalization duration on recovery remains uncertain. Long-term randomized studies are needed to further investigate the relationship between NGT use, weight gain during hospitalization, and recovery.

The broad age range (9 to 20 years) encompasses substantial variability in growth and pubertal development that cannot be fully captured by the BMI z-score despite adjustment for age. Differences in growth velocity, pubertal development, and interpretation of BMI z-scores across this age range may have influenced the measured weight trajectories despite statistical adjustment for age.

Interpretation of subgroup analyses (Table 4) should remain cautious because the ARFID cohort was small, variability was substantial, and the statistical power for multivariable subgroup analyses was limited. Although the mixed-effects model demonstrated a good overall fit (conditional R^2^ = 0.92) and was supported by AIC, BIC, and likelihood ratio tests, potential instability and overfitting should be acknowledged [47]. ARFID subtype information was not systematically collected, limiting interpretation of how subtype-specific characteristics may have influenced the nutritional rehabilitation outcomes. Accordingly, the findings should be viewed as exploratory and hypothesis generating until replicated in larger cohorts.

Lastly, psychological measures were not collected; therefore, patient-reported distress related to NGT placement, enteral feeding, and progression through nutritional rehabilitation could not be assessed. FBT has been adapted to treat ARFID and is used in this refeeding protocol to help support patients through nutritional rehabilitation [48,49], but it does not fully address the sensory or phobia needs of children with ARFID. Incorporating assessment by a sensory feeding specialist, such as an occupational therapist or speech-language pathologist, may be helpful in the treatment of patients with ARFID [16]. Further research is needed to assess how specific ARFID subtypes influence tolerance of refeeding and how feeding therapists are best incorporated into inpatient stabilization programs.

## 5. Conclusions

Evidence-based nutritional rehabilitation approaches for ARFID remain limited, and many inpatient programs continue to adapt treatment strategies developed for AN. In this cohort, youth hospitalized with ARFID achieved weight outcomes comparable to patients with AN/AAN but required a substantially higher use of enteral nutrition support. Patients with ARFID requiring NGT supplementation demonstrated higher nutritional impairment at presentation, suggesting likely confounding by indication. These findings support the need for ARFID-specific medical stabilization pathways and prospective multicenter studies to better define the role of enteral nutrition during inpatient nutritional rehabilitation.

## Figures and Tables

**Table 1:** Sample characteristics.

	Overall	Anorexia Nervosa (AN) and Atypical AN	Avoidant/Restrictive Food Intake Disorder (ARFID)	*p*-Value
*n*	160	126	34	
Demographics				
Age at admission (mean standard deviation (SD))	14.74 (2.18)	14.90 (1.84)	14.15 (3.10)	0.075
Sex = Male (%)	21 (13.1)	15 (11.9)	6 (17.6)	0.553
Race (%)				0.109
White, non-Hispanic	127 (82.5)	100 (82.6)	27 (81.8)	
Black, non-Hispanic	11 (7.1)	6 (5.0)	5 (15.2)	
Asian, non-Hispanic	3 (1.9)	3 (2.5)	0 (0.0)	
Hispanic	13 (8.4)	12 (9.9)	1 (3.0)	
Length of stay, days (mean (SD))	14.78 (9.11)	14.40 (9.07)	16.15 (9.26)	0.324
Discharge location = Residential eating disorder facility/Inpatient psychiatric unit (%)	25 (15.6)	20 (15.9)	5 (14.7)	1.000
Weight, BMI, and Nutrition				
Weight (admission), kilograms (kg) (mean (SD))	44.09 (11.07)	45.22 (9.39)	39.89 (15.26)	0.012
Weight (discharge), kg (mean (SD))	46.98 (10.77)	48.25 (8.87)	42.26 (15.22)	0.004
Weight change (mean (SD))	2.89 (2.06)	3.04 (2.15)	2.37 (1.60)	0.094
BMI (admission) (mean (SD))	17.22 (3.28)	17.43 (2.95)	16.46 (4.27)	0.126
BMI (discharge) (mean (SD))	18.41 (3.07)	18.63 (2.69)	17.57 (4.15)	0.074
BMI change (mean (SD))	1.18 (0.85)	1.20 (0.88)	1.11 (0.76)	0.588
Malnutrition (%)				0.74
Not malnourished	16 (10.4)	14 (11.7)	2 (5.9)	
Mild	16 (10.4)	13 (10.8)	3 (8.8)	
Moderate	27 (17.5)	20 (16.7)	7 (20.6)	
Severe	95 (61.7)	73 (60.8)	22 (64.7)	
NGT				
NGT (*N* (%))	26 (16.2)	12 (9.5)	14 (41.2)	*p* < 0.001
NGT Category (%)				0.098
Used for post meal replacement only and removed after each use	16 (61.5)	10 (83.3)	6 (42.9)	
Used for overnight feeds only and left in place	1 (3.8)	0 (0.0)	1 (7.1)	
Used for overnight feeds and post meal replacement and left in place	9 (34.6)	2 (16.7)	7 (50.0)	
Medications				
Laxatives (*N* (%))	73 (45.6)	59 (46.8)	14 (41.2)	0.694
Supplements (*N* (%))	149 (93.1)	118 (93.7)	31 (91.2)	0.901
Antihistamines (*N* (%))	107 (66.9)	79 (62.7)	28 (82.4)	0.051
Selective Serotonin Reuptake Inhibitor (*N* (%))	109 (68.1)	84 (66.7)	25 (73.5)	0.579
Antipsychotics (*N* (%))	26 (16.2)	20 (15.9)	6 (17.6)	1.000
Anxiolytics (*N* (%))	8 (5.0)	7 (5.6)	1 (2.9)	0.859
Antiemetics (*N* (%))	13 (8.1)	9 (7.1)	4 (11.8)	0.602
Stimulants (*N* (%))	1 (0.6)	0 (0.0)	1 (2.9)	0.481
Acid reducers (*N* (%))	27 (16.9)	17 (13.5)	10 (29.4)	0.052

Patient demographics, BMI and nutrition data, NGT use, and medications continued at discharge stratified by patient group: AN/AAN and ARFID. BMI: body mass index, NGT: nasogastric tube, AN: anorexia nervosa, AAN: atypical anorexia nervosa, ARFID: avoidant/restrictive food intake disorder, SD: standard deviation, kg: kilograms.

**Table 2:** Patient comorbidities.

	Overall	Anorexia Nervosa (AN) and Atypical AN (AAN) *N* (%)	Avoidant/Restrictive Food Intake Disorder (ARFID) *N* (%)	χ^2^ *p*-Value
*n*	160	126	34	
Depression/Major depressive disorder	87 (54.4)	81 (64.3)	6 (17.6)	*p* < 0.001
Anxiety/Generalized anxiety disorder	124 (77.5)	94 (74.6)	30 (88.2)	0.145
Obsessive compulsive disorder	11 (6.9)	9 (7.1)	2 (5.9)	1.00
Bipolar disorder	2 (1.2)	2 (1.6)	0 (0.0)	1.00
Autism spectrum disorder	1 (0.6)	0 (0.0)	1 (2.9)	0.481
Victim of bullying	3 (1.9)	2 (1.6)	1 (2.9)	1.00
History of trauma and/or abuse	7 (4.4)	5 (4.0)	2 (5.9)	0.991
Attention-deficit/hyperactivity disorder	10 (6.2)	8 (6.3)	2 (5.9)	1.00
Phobia (such as fear of vomiting or choking)	14 (8.8)	2 (1.6)	12 (35.3)	*p* < 0.001
Constipation (with or without encopresis)	14 (8.8)	11 (8.7)	3 (8.8)	1.00
Irritable bowel syndrome or functional abdominal pain syndrome	1 (0.6)	0 (0.0)	1 (2.9)	0.481
Nausea and vomiting	3 (1.9)	1 (0.8)	2 (5.9)	0.219
Substance use or dependence: marijuana	6 (3.8)	4 (3.2)	2 (5.9)	0.819
Diabetes mellitus	3 (1.9)	1 (0.8)	2 (5.9)	0.219
Other	20 (12.5)	14 (11.1)	6 (17.6)	0.465
None	4 (2.5)	4 (3.2)	0 (0.0)	0.665

Comorbid conditions present on admission for medical stabilization stratified by patient group: AN/AAN, and ARFID. AN/AAN: anorexia nervosa, atypical anorexia nervosa, ARFID: avoidant/restrictive food intake disorder.

**Table 3:** Generalized linear mixed effects modeling (GLMM) analysis.

	Estimate (95% CI)	df	t Value	*p*-Value
(Intercept)	0.76 (0.35–1.16)	147.52	3.54	*p* < 0.001
Age at admission, years	−0.05 (−0.08–−0.03)	147.4	−3.85	*p* < 0.001
Sex				
Female	Ref.			
Male	−0.01 (−0.17–0.14)	148.35	−0.18	0.86
BMI z-score at admission	−0.04 (−0.08–−0.01)	159.34	−2.22	0.03
Time from admission, weeks	0.19 (0.17–0.21)	2053.24	21.37	*p* < 0.001
NG tube				
No	Ref.			
Yes	0.14 (−0.06–0.35)	160.14	1.32	0.19
Patient group				
AN	Ref.			
ARFID	0.04 (−0.14–0.22)	167.22	0.4	0.69
Depression/Major depressive disorder (MDD)	0.02 (−0.09–0.14)	147.06	0.39	0.69
Anxiety/Generalized anxiety disorder (GAD)	−0.06 (−0.18–0.06)	148.48	−0.94	0.35
Obsessive compulsive disorder (OCD)	0.05 (−0.15–0.26)	146.67	0.51	0.61
Phobia (such as fear of vomiting or choking)	−0.04 (−0.26–0.18)	147.87	−0.34	0.73
Time × BMI z-score at admission	−0.09 (−0.10–−0.09)	2066.91	−31.63	*p* < 0.001
Time × Group	−0.06 (−0.10–−0.01)	2044.11	−2.54	0.01
Time × NGT	−0.15 (−0.18–−0.13)	2055.42	−13.87	*p* < 0.001
NGT × Group	−0.24 (−0.56–0.08)	169.5	−1.41	0.16
Time × NGT × Group	0.13 (0.08–0.19)	2051.72	4.66	*p* < 0.001

Model summary of GLMM analysis of weight change (BMI z-scores) over time, which shows an interaction between patient group (AN/AAN versus ARFID), NGT placement and time. GLMM: generalized linear mixed effects modeling, BMI: body mass index, AN/AAN: anorexia nervosa, atypical anorexia nervosa, ARFID: avoidant/restrictive food intake disorder, NGT: nasogastric tube.

**Table 4:** Aggregate data comparison of the avoidant/restrictive food intake (ARFID) groups.

	Overall	No NGT	NGT	*p*-Value
*n*	34	20	14	
Age at admission (mean (standard deviation (SD)))	14.15 (3.10)	15.20 (2.93)	12.64 (2.76)	0.015
Sex = Male (%)	6 (17.6)	3 (15.0)	3 (21.4)	0.979
Race (%)				
White, non-Hispanic	27 (81.8)	15 (75.0)	12 (92.3)	
Black, non-Hispanic	5 (15.2)	4 (20.0)	1 (7.7)	
Asian, non-Hispanic	0 (0.0)	0 (0.0)	0 (0.0)	
Hispanic	1 (3.0)	1 (5.0)	0 (0.0)	
Length of stay, days (mean (SD))	16.15 (9.26)	11.80 (3.27)	22.36 (11.46)	*p* < 0.001
Discharge location = Residential eating disorder facility/Inpatient psychiatric unit (%)	5 (14.7)	2 (10.0)	3 (21.4)	0.664
Weight (admission), kilograms (kg) (mean (SD))	39.89 (15.26)	46.44 (15.77)	30.53 (8.23)	0.002
Weight (discharge), kg (mean (SD))	42.26 (15.22)	48.55 (15.98)	33.28 (8.19)	0.003
Weight change (mean (SD))	2.37 (1.60)	2.10 (1.03)	2.75 (2.17)	0.25
BMI (admission) (mean (SD))	16.46 (4.27)	17.98 (4.74)	14.29 (2.21)	0.011
BMI z-score (admission) (mean (SD))	−1.86 (1.76)	−1.34 (1.67)	−2.61 (1.66)	0.037
BMI (discharge) (mean (SD))	17.57 (4.15)	18.89 (4.74)	15.68 (2.09)	0.024
BMI z-score (discharge) (mean (SD))	−1.10 (1.40)	−0.83 (1.51)	−1.47 (1.20)	0.197
BMI change (mean (SD))	1.11 (0.76)	0.92 (0.51)	1.39 (0.97)	0.072
BMI z-score change (mean (SD))	0.76 (0.76)	0.50 (0.41)	1.13 (0.99)	0.015
Malnutrition (%)				0.858
Not malnourished	2 (5.9)	1 (5.0)	1 (7.1)	
Mild	3 (8.8)	2 (10.0)	1 (7.1)	
Moderate	7 (20.6)	5 (25.0)	2 (14.3)	
Severe	22 (64.7)	12 (60.0)	10 (71.4)	

Description of patients with ARFID and comparison of BMI measures and length of stay stratified by NGT use (*t*-tests). ARFID: avoidant/restrictive food intake disorder; BMI: body mass index; NGT: nasogastric tube; standard deviation: SD; kg: kilograms.

## Data Availability

The datasets generated and/or analyzed during the current study are not publicly available because of participant privacy restrictions but may be available from the corresponding author on reasonable request and with institutional approval.

## Abbreviations

The following abbreviations are used in this manuscript.

ARFID	Avoidant/restrictive food intake disorder
AN	Anorexia nervosa
NGT	Nasogastric tube
AAN	Atypical anorexia nervosa
BMI	Body mass index
DSM-5	Diagnostic and Statistical Manual-5
FBT	Family-based treatment
CBT	Cognitive behavioral therapy
GLMM	Generalized linear mixed effects modeling
R^2^c	Conditional R^2^
SSRIs	Selective serotonin reuptake inhibitors
